# Haploinsufficiency of the autism-associated *Shank3 *gene leads to deficits in synaptic function, social interaction, and social communication

**DOI:** 10.1186/2040-2392-1-15

**Published:** 2010-12-17

**Authors:** Ozlem Bozdagi, Takeshi Sakurai, Danae Papapetrou, Xiaobin Wang, Dara L Dickstein, Nagahide Takahashi, Yuji Kajiwara, Mu Yang, Adam M Katz, Maria Luisa Scattoni, Mark J Harris, Roheeni Saxena, Jill L Silverman, Jacqueline N Crawley, Qiang Zhou, Patrick R Hof, Joseph D Buxbaum

**Affiliations:** 1 Seaver Autism Center for Research and Treatment, Mount Sinai School of Medicine, New York, NY 10029, USA; 2 Department of Psychiatry, Mount Sinai School of Medicine, New York, NY 10029, USA; 3 Department of Neuroscience, Mount Sinai School of Medicine, New York, NY 10029, USA; 4 Department of Neurology, Mount Sinai School of Medicine, New York, NY 10029, USA; 5 Department of Genetics and Genomic Sciences, Mount Sinai School of Medicine, New York, NY 10029, USA; 6 Laboratory of Behavioral Neuroscience, National Institute of Mental Health, Bethesda, MD 20892-3730, USA; 7 Istituto Superiore di Sanità, Rome, Italy; 8 Genentech, South San Francisco, CA 94080, USA

## Abstract

**Background:**

SHANK3 is a protein in the core of the postsynaptic density (PSD) and has a critical role in recruiting many key functional elements to the PSD and to the synapse, including components of α-amino-3-hydroxyl-5-methyl-4-isoxazole-propionic acid (AMPA), metabotropic glutamate (mGlu) and *N*-methyl-D-aspartic acid (NMDA) glutamate receptors, as well as cytoskeletal elements. Loss of a functional copy of the *SHANK3 *gene leads to the neurobehavioral manifestations of 22q13 deletion syndrome and/or to autism spectrum disorders. The goal of this study was to examine the effects of haploinsufficiency of full-length *Shank3 *in mice, focusing on synaptic development, transmission and plasticity, as well as on social behaviors, as a model for understanding *SHANK3 *haploinsufficiency in humans.

**Methods:**

We used mice with a targeted disruption of *Shank3 *in which exons coding for the ankyrin repeat domain were deleted and expression of full-length Shank3 was disrupted. We studied synaptic transmission and plasticity by multiple methods, including patch-clamp whole cell recording, two-photon time-lapse imaging and extracellular recordings of field excitatory postsynaptic potentials. We also studied the density of GluR1-immunoreactive puncta in the CA1 stratum radiatum and carried out assessments of social behaviors.

**Results:**

In *Shank3 *heterozygous mice, there was reduced amplitude of miniature excitatory postsynaptic currents from hippocampal CA1 pyramidal neurons and the input-output (I/O) relationship at Schaffer collateral-CA1 synapses in acute hippocampal slices was significantly depressed; both of these findings indicate a reduction in basal neurotransmission. Studies with specific inhibitors demonstrated that the decrease in basal transmission reflected reduced AMPA receptor-mediated transmission. This was further supported by the observation of reduced numbers of GluR1-immunoreactive puncta in the stratum radiatum. Long-term potentiation (LTP), induced either with θ-burst pairing (TBP) or high-frequency stimulation, was impaired in *Shank3 *heterozygous mice, with no significant change in long-term depression (LTD). In concordance with the LTP results, persistent expansion of spines was observed in control mice after TBP-induced LTP; however, only transient spine expansion was observed in *Shank3 *heterozygous mice. Male *Shank3 *heterozygotes displayed less social sniffing and emitted fewer ultrasonic vocalizations during interactions with estrus female mice, as compared to wild-type littermate controls.

**Conclusions:**

We documented specific deficits in synaptic function and plasticity, along with reduced reciprocal social interactions in *Shank3 *heterozygous mice. Our results are consistent with altered synaptic development and function in *Shank3 *haploinsufficiency, highlighting the importance of Shank3 in synaptic function and supporting a link between deficits in synapse function and neurodevelopmental disorders. The reduced glutamatergic transmission that we observed in the *Shank3 *heterozygous mice represents an interesting therapeutic target in *Shank3*-haploinsufficiency syndromes.

## Background

### The Shank protein family

**Figure 1:**
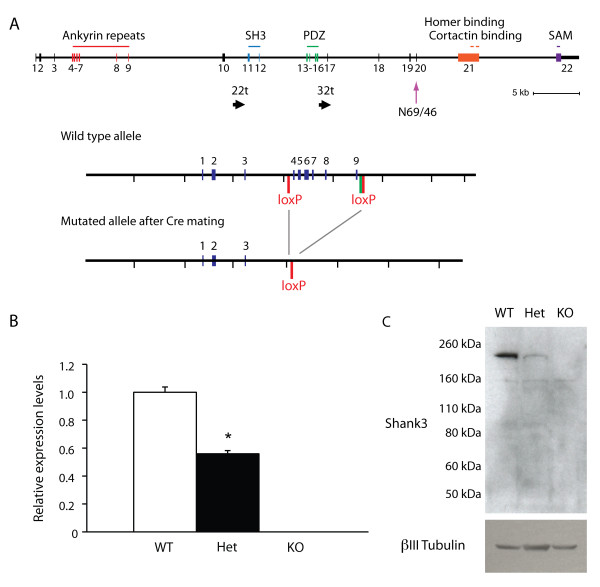
**Reduced expression of Shank3 in *Shank3*-deficient mice**. **(A) **Targeting strategy. *Top*: The genomic structure of *Shank3 *is shown. The position of the 22t and 32t transcripts are also shown, as is the epitope for antibody N69/46. *Bottom*: The location of the loxP sites introduced for targeting are shown before and after the activity of the recombinase. The green bar in the wild-type allele indicates the FRT site remaining after deletion of the selection cassette. **(B) **Expression of Shank3 mRNA. Brain-derived mRNA from wild-type, heterozygous and knockout mice were subjected to quantitative polymerase chain reaction (qPCR) assay for Shank3 mRNA using probes across exons 6 and 7 with normalization against reference genes. **(C) **Expression of Shank3 in postsynaptic density (PSD) fractions. PSD fractions from wild-type, heterozygous and knockout mice were subjected to immunoblotting with antibody N69/46 to Shank3. The migration of molecular weight markers is shown on the left (in kilodaltons) and an immunoblot for βIII-tubulin as a loading control is shown below. WT, wild-type mice; Het, *Shank3 *heterozygous mice; KO, homozygous knockout mice. *P < 0.0003.

Shank proteins, which include Shanks 1, 2 and 3, were first identified in a yeast two-hybrid assay using the guanylate kinase-associated protein (GKAP, also called SAPAP1, DLGAP1 and DAP-1) as bait [1]. GKAP is a PSD-95-binding protein that forms an important component of the postsynaptic density (PSD), where protein-protein interactions between scaffolding proteins and receptors are a key mechanism in assembling a functional synapse [2]. Shanks are highly enriched in the PSD and contain five domains for protein-protein interactions, including an ankyrin repeat domain, an Src homology 3 (SH3) domain, a PSD-95/discs large/zonula occludens-1 (PDZ) domain, several proline-rich regions and a C-terminal sterile α-motif (SAM) domain [3] (Figure 1). The PDZ domain mediates the interaction of Shanks with the COOH terminus of several different proteins, including GKAP [1,4], G protein-coupled receptors [5,6] and the COOH terminus of group I metabotropic glutamate receptors (mGluRs) [7]. GKAP mediates the binding of Shanks to *N*-methyl-D-aspartic acid (NMDA) and α-amino-3-hydroxyl-5-methyl-4-isoxazole-propionic acid (AMPA) receptors [8]. In addition, the SAM domain interacts with GKAP [7], while proline-rich regions of the Shank proteins bind the mGluR-binding protein Homer [7], actin-binding protein Abp-1 [9,10] and cortactin (cortical actin-binding protein) [11], which promotes polymerization of the actin cytoskeleton, an important modulator of long-term synaptic plasticity [12]. Shanks interact directly with AMPA receptors through the SH3 domain [13]. Interaction of the ankyrin repeats of Shanks with α-fodrin may mediate calmodulin-mediated processes after synaptic stimulation through interactions with actin and calmodulin [14]. Sharpin can also interact with the Shanks [15] and may be involved in activity-dependent modification in dendritic spines.

### Shank proteins in synaptic biology

There are ~300 individual Shank molecules in a single postsynaptic site, representing ~5% of the total protein molecules and total protein mass in the site [16]. It would therefore not be surprising that alteration in Shank expression could profoundly affect synaptic morphology and function. Such a crucial role for Shank levels in synaptic function is supported by the observation that overexpression of *Shank1 *led to increased spine size in neurons in culture [17]. Additional studies showed that knockout of *Shank1 *leads to a decrease in spine number and spine and PSD size, decreased levels of GKAP and Homer, and reduced basal synaptic transmission [18]. Furthermore, inhibition of *Shank3 *expression has been shown to reduce numbers of spines in hippocampal neurons in culture and, conversely, when *Shank3 *was introduced into aspiny cerebellar neurons *in vitro*, the neurons developed spines with functional glutamatergic synapses expressing NMDA, AMPA and mGlu receptors [19].

### Haploinsufficiency of *SHANK3 *in neurodevelopmental syndromes

Chromosome 22q13 deletion syndrome (22q13DS, also called Phelan-McDermid syndrome) was first described in the early 1990s (reviewed in [20]). Affected individuals show global developmental delays with absent or severely delayed expressive speech, and they may have autism spectrum disorders (ASDs). Careful analysis of the extent of the deletion in dozens of independent cases indicated the presence of a small "critical region" encompassing *SHANK3 *[21,22], providing the first line of evidence that a dysfunction in SHANK3 may be responsible for the neurobehavioral aspects of 22q13DS. The second line of evidence was the demonstration of a recurrent breakpoint in *SHANK3 *in some cases with 22q13DS [23,24], which led these authors to conclude that disruption of this single gene might be sufficient to cause 22q13DS. Third, there are translocations in *SHANK3 *that lead to 22q13DS [25]. Finally, several recent studies demonstrated that even *de novo *point mutations in *SHANK3 *can produce the entirety of neurodevelopmental symptoms of 22q13DS, including global developmental delay, absent or severely delayed expressive speech, and ASDs [26-28]. Neurobehavioral phenotypes associated with mutation or deletion of *SHANK3 *are here referred to as *SHANK3*-haploinsufficiency syndromes and, as noted above, can be associated with ASDs. Interestingly, overexpression of *SHANK3 *may also result in an ASD as evidenced by reports of Asperger syndrome in an individual with three copies of the *SHANK3 *locus [27]. Recently, *de novo *missense and nonsense mutations in *SHANK3 *have been described in atypical schizophrenia (with mild to moderate intellectual disability, early onset and dysmorphic features [29]). Mutations in the highly related gene *SHANK2 *have also recently been associated with ASDs and/or intellectual disability [29-31].

In the current study, we characterized mice with a targeted disruption of *Shank3 *as a model for *SHANK3*-haploinsufficiency syndromes. We focused on synaptic biology and synaptic function as the most proximal target for altered Shank3 expression. We also examined social interaction and social communication in *Shank3 *heterozygotes and their wild-type littermates. The results are consistent with an important role for SHANK3 in synaptic function and plasticity and implicate specific pathways as potential therapeutic targets for *SHANK3*-haploinsufficiency syndromes.

## Methods

### Generation of mice with a disruption of the *Shank3 *gene

Animal procedures were approved by the Mount Sinai School of Medicine and the National Institute of Mental Health Animal Care and Use committees. We made use of gene targeting in Bruce4 C57BL/6 embryonic stem (ES) cells [32] to generate a mouse line that has loxP sites inserted before exon 4 and after exon 9 (encoding the ankyrin repeats), with the selection cassette (flanked by FRT sites) excised by FLP recombinase. This floxed strategy was chosen to allow us the option of doing conditional (region-specific) knockouts if needed. C57BL/6 was used as the chosen embryonic stem cell line because of the more robust social and cognitive abilities in this line as compared to many of the 129-derived ES lines. For all studies reported here, the floxed allele was first excised by crossing with a CMV-Cre transgenic line (again on a C57BL/6 background) that has ubiquitous Cre expression and a line maintained with a deletion of exons 4 through 9. This *Shank3*-deficient line was carried forward by crossing heterozygotes with the C57BL/6 strain to maintain a pure C57 background suitable for electrophysiology and behavioral analyses.

### Quantitative polymerase chain reaction

RNA was extracted from brain cortex using the RNeasy Mini Kit (Qiagen, Valencia, CA, USA) according to the manufacturer's instructions. cDNA was synthesized with the High Capacity cDNA Reverse Transcription Kit (Applied Biosystems, Carlsbad, CA, USA). The universal probe library (UPL) system (Roche, Indianapolis, IN, USA) was used to perform quantitative polymerase chain reaction (qPCR). Primers located in exons 6 and 7 of Shank3 (NM_021423) were designed using ProbeFinder Software (Roche). Three reference genes (*Actb*, *Gapd *and *Rpl13a*) were used for normalization, and relative expression levels were calculated using qBase software [33], now available from Biogazelle (Ghent, Belgium). Primer sequences and UPL probe numbers were Shank3, forward tggttggcaagagatccat, reverse ttggccccatagaacaaaag, #1; Actb, forward ggatgcagaaggagattactgc, reverse ccaccgatccacacagagta, #63; Gapd, Fw gccaaaagggtcatcatctc, reverse cacacccatcacaaacatgg, #29; Rpl 13a, forward tccctgctgctctcaagg, reverse gccccaggtaagcaaactt, #41. Unpaired *t*-tests were used for group comparisons.

### Immunoblotting

PSD fractions were prepared as follows. Hemibrains of wild-type, heterozygous and homozygous *Shank3 *mice were homogenized in 2-[4-(2-hydroxyethyl)piperazin-1-yl]ethanesulfonic acid (HEPES)-A containing 4 mM HEPES, pH 7.4, 0.32 M sucrose, Protease Inhibitor Cocktail and PhoSTOP Phosphatase Inhibitor Cocktail (both from Roche). Nuclear fractions were precipitated by centrifuging twice at 700 *g *for 15 min, and the resulting supernatants were further centrifuged at 21,000 *g *for 15 min. The precipitates were resuspended in HEPES-B containing 4 mM HEPES, pH 7.4, Protease Inhibitor Cocktail and PhoSTOP Phosphatase Inhibitor Cocktail, homogenized and rotated at 4°C for 1 hour. The lysates were centrifuged at 32,000 *g *for 20 min and washed twice with HEPES-C containing 50 mM HEPES, pH 7.4, 0.5% Triton X-100, Protease Inhibitor Cocktail and PhoSTOP Phosphatase Inhibitor Cocktail. Finally, postsynaptic density fractions were resuspended in HEPES-C containing 1.8% sodium dodecyl sulfate (SDS) and 2.5 M urea. Fifty-two μg of each PSD fraction were loaded to 4-12% SDS-polyacrylamide gel electrophoresis (PAGE gel (Invitrogen, Carlsbad, CA, USA), transferred to polyvinylidene fluoride membrane and immunoblotted with either the N69/46 anti-Shank3 antibody directed against an epitope downstream of the PDZ domain (UC Davis/NIH NeuroMab Facility, Davis, CA) or the anti-ProSAP2 anti-Shank3 antibody directed against the last 100 amino acids of Shank3 (Millipore, Billerica, MA, USA). For βIII tubulin, the membrane was stripped and immunoblotted with an anti-βIII tubulin antibody (Abcam, Cambridge, MA, USA).

### Hippocampal slice electrophysiology

#### Whole cell recording, two-photon time-lapse imaging and analysis

Methods of recording, imaging and analysis were carried out according to our previously published protocols [34,35]. All experiments were conducted on CA1 pyramidal cells at 32°C in acute slices taken from *Shank3 *heterozygous mice and wild-type littermates. Spines were visualized using calcein contained in the patch pipette, making use of a two-photon laser scanning system modified from Olympus Fluoview FV 300 driven by a Chameleon two-photon laser (Coherent, Santa Clara, CA, USA). Baseline synaptic responses were evoked using a glass pipette positioned ~20 μm away from the imaged spines and recorded at the soma. Long-term potentiation (LTP) was induced with a θ-burst pairing (TBP) protocol in which two trains of θ-burst stimuli (each train, separated by 20 s, consisted of five bursts at 5 Hz, and each burst contained five pulses at 100 Hz) were paired with brief, small postsynaptic depolarization. Volume analysis of individual spines was performed as detailed previously [34]. Briefly, the integrated fluorescence intensity inside a spine head was measured for individual spines at different time points and normalized to the fluorescence intensity of the dendrites from the same image stack to correct for potential changes in excitation [36]. Spine volume in the θ-burst stimulation (TBS) experiments was also calculated using the Rayburst algorithm in NeuronStudio software (available from the Computational Neurobiology and Imaging Center, Mount Sinai School of Medicine, New York, NY, USA) following deconvolution of the data [37-39], and we obtained similar results using either approach.

#### Extracellular recordings

Hippocampal slices (350 μm thick) were prepared from 4- to 6-week-old heterozygous mice and their wild-type littermate controls. Slices were perfused with Ringer's solution containing (in mM): NaCl, 125.0; KCl, 2.5; MgSO_4_, 1.3; NaH_2_PO_4_, 1.0; NaHCO_3_, 26.2; CaCl_2_, 2.5; and glucose, 11.0. The Ringer's solution was bubbled with 95% O_2 _and 5% CO_2 _at 32°C during extracellular recordings (electrode solution: 3 M NaCl). Slices were maintained for 1 hour prior to establishment of a baseline of field excitatory postsynaptic potentials (fEPSPs) recorded from stratum radiatum in area CA1, evoked by stimulation of the Schaffer collateral-commissural afferents (100-μs pulses every 30 s) with bipolar tungsten electrodes placed into area CA3 [40]. Test stimulus intensity was adjusted to obtain fEPSPs with amplitudes that were one-half of the maximal response. The EPSP initial slope (mV/ms) was determined from the average waveform of four consecutive responses. Input-output (I/O) curves were generated by plotting the fEPSP slope versus fiber volley amplitude in low-Mg^2+ ^(0.1 mM) solution. AMPA receptor-mediated and NMDA receptor-mediated I/O relationships were measured in the presence of 2-amino-5-phosphonopentanoic acid (APV; 50 μM) and 6-cyano-7-nitroquinoxaline-2,3-dione (CNQX; 100 μM), respectively (Sigma, St. Louis, MO, USA).

Paired-pulse responses were measured with an interstimulus interval (ISI) of 50 ms and are expressed as the ratio of the average responses to the second stimulation pulse (FP2) to the first stimulation pulse (FP1). LTP was induced by either a high-frequency stimulus (four trains of 100-Hz, 1-s stimulations separated by 5 min) or TBS (15 bursts of four pulses at 100 Hz separated by 200 ms). To induce long-term depression (LTD), Schaffer collaterals were stimulated by low-frequency stimulation (LFS; 900 pulses at 1 Hz, 15 min) or by a paired-pulse low-frequency stimulation (PP-LFS; 1 Hz for 20 min, 50-ms interstimulus interval [41]) to induce mGlu receptor-dependent LTD. Data were expressed as means ± SD, and statistical analyses were performed using analysis of variance (ANOVA) or Student's *t*-test, with significance set at an α level of 0.05.

### Measurement of GluR1-immunoreactive puncta

#### Immunohistochemistry

Three-month-old animals were anesthetized with 250 μl of 15% chloral hydrate and perfused transcardially with 1% paraformaldehyde for 1 min followed by 4% paraformaldehyde for a total of 13 min. The brains were then removed, hemisected, cut in 50-μm-thick sections using a Leica VT1000S Vibratome (Vibratome, Bannockburn, IL, USA) and subsequently stored in phosphate-buffered saline (PBS) until use. Sections were incubated at 37°C for 5 min, followed by incubation in acidified pepsin (1 ml in a 0.2 N HCl solution) for 6.5 min. The tissue was then washed at room temperature in PBS-B (3 × 20 min) and incubated in a 0.3% Triton X-100, 0.5% bovine serum albumin (BSA), 5% normal goat serum for 1 h on an orbital shaker. The blocking step was followed by overnight incubation in the primary antibody (rabbit polyclonal antiglutamate receptor 1 AB1504; Millipore, Billerica, MA, USA), which was made in blocking solution at the appropriate dilution (1 μg/ml). The tissue sections were then washed in PBS-B (5 × 5 min) and incubated in secondary antibody (goat antirabbit Alexa Fluor 488, Invitrogen) in a 2% BSA and 0.3% Triton PBS-B solution at the appropriate dilution (1:400) for 1 h at room temperature on an orbital shaker. Finally, the tissue was washed in PBS-B (3 × 5 min), stained with 4",6"-diamino-2-phenylindole-2HCl (DAPI) and mounted on charged Aqua ColorFrost slides using Vectashield mounting medium (Vector Laboratories, Burlingame, CA, USA).

#### Tissue sampling

To quantify puncta, we used a systematic random sampling approach whereby a 1:6 series of sections were stained, the stratum radiatum of the CA1 was contoured using SteroInvestigator (MBF Bioscience, Williston, VT, USA) and the sampling sites were determined using an optical fractionator with the size of the grid set at 18 μm^2 ^(the dimension of the confocal image stacks to be later sampled with a ×100 lens objective), at a digital zoom of 5 on a Zeiss LSM510 META confocal microscope (Zeiss, Oberkochen, Germany). The trace of the contoured area with an optical fractionator sampling grid placed on it was used as a guide to obtain confocal image stacks of the above-mentioned dimensions that were 100 μm apart from each other (i.e., the size of the probe).

#### Confocal imaging and puncta quantification

The fluorescent puncta were visualized under a ×100 oil immersion objective (1.4 numerical aperture) in a series of Z-stacks using an Argon/2 laser (488 nm wavelength) at 50% output (tube current of 6.4 A and maximum power of 30 mW), with a collection band pass spectrum of 505-550 nm (with the following laser and microscope settings: image frame size of 512 × 512, 1 Airy unit, refractive index correction of 0.9144 and Z stack interval of 0.1 μm (*x*,*y *pixel size = 0.05 μm)) and their intensity, number and size quantified. These settings were optimized during pilot studies and held constant throughout the study.

The resultant stacks were then deconvolved using AutoDeblur 1.4.1 (Media Cybernetics, Bethesda, MD, USA), using an adaptive point-spread function (PSF) deconvolution method with a theoretical PSF, and then analyzed with custom Vamp2D software [42] that reliably calculates the size of individual puncta on the basis of three-dimensional estimates and circumvents the problem of object superimposition found with more traditional methods that collapse the stacks into two-dimensional projections. The relative density of puncta was then calculated per cubic micrometer, and the differences between groups were assessed using the nonparametric Mann-Whitney *U *test.

### Behavioral analyses

*Shank3 *wild-type and heterozygote breeding pairs were imported from Mount Sinai School of Medicine to the National Institute of Mental Health. Mice were maintained by breeding C57BL/6 wild-type mice with *Shank3 *heterozygotes and housed in a conventional temperature- and humidity-controlled vivarium. Littermates were housed by sex in mixed genotype groups of two to four per cage on a 12:12-h circadian cycle with lights on at 0600. Behavioral experiments were conducted between 1000 and 1600 in dedicated testing rooms.

Developmental milestones were tested across postnatal days 2-14, including measures of body weight, body length, tail length, pinnea detachment, eye opening, incisor eruption, fur development, righting reflex, negative geotaxis, cliff avoidance, grasping reflex, auditory startle, bar holding, level screen and vertical screen as previously described [43,44]. In addition, the mice were evaluated in a standard, automated three-chambered social approach task as previously described [44].

Male-female social interactions were evaluated in a 5-min test session as previously described [43,45], with the exception that subject males were group-housed and individually tested in clean cages with clean litter. Each of the 12 wild-type and 14 heterozygous male subject mice, ages 2.5-4 months, was paired with a different unfamiliar estrus C57BL/6J female. A digital closed-circuit television camera (Panasonic, Secaucus, NJ, USA) was positioned horizontally 30 cm from the cage. An ultrasonic microphone (Avisoft UltraSoundGate condenser microphone capsule CM15; Avisoft Bioacoustics, Berlin, Germany) was mounted 20 cm above the cage. Sampling frequency for the microphone was 250 kHz, and the resolution was 16 bits. The entire apparatus was contained in a sound-attenuating environmental chamber (ENV-018V; Med Associates, St. Albans, VT, USA) illuminated by a single 25-Watt red light. Videos from the male subjects were subsequently scored by an investigator uninformed of the subject's genotype on measures of nose-to-nose sniffing, nose-to-anogenital sniffing and sniffing of other body regions, using Noldus Observer software (Noldus Information Technology, Leesburg, VA, USA) as previously described. Ultrasonic vocalizations were played back and spectrograms were displayed using Avisoft software [43,45]. Ultrasonic vocalizations were identified manually by two highly trained investigators blinded to genotype information, and summary statistics were calculated using the Avisoft package. Interrater reliability was 95%. Data were analyzed using an unpaired Student's *t*-test.

Olfactory habituation/dishabituation testing was conducted in male and female *Shank3 *wild-type and heterozygous mice ages 2.5-4 months using methods previously described [44,46,47]. Nonsocial and social odors were presented on a series of cotton swabs inserted into the home cage sequentially, each for 2 min, in the following order: water, water, water (distilled water); almond, almond, almond (1:100 dilution almond extract); banana, banana, banana (1:100 dilution artificial banana flavoring); social 1, social 1, social 1 (swiped from the bottom of a cage housing unfamiliar sex-matched B6 mice); and social 2, social 2, social 2 (swiped from the bottom of a second cage housing a different group of unfamiliar sex-matched 129/SvImJ mice). One-way repeated measures ANOVA was performed within each genotype for each set of habituation events and each dishabituation event, followed by a Tukey *post hoc *test.

## Results

### Generation and characterization of a *Shank3*-deficient mouse

We made use of gene targeting in Bruce4 C57BL/6 embryonic stem (ES) cells [32] to generate a mouse line that has loxP sites inserted before exon 4 and after exon 9 (Figure 1A). For all studies reported here, the floxed allele was excised and a line was maintained with a deletion of exons 4 through 9. This line, which completely deletes the ankyrin repeat domains of Shank3, produced wild-type (+/+), heterozygous (+/-) and knockout (-/-) animals with Mendelian frequencies from heterozygote-heterozygote crosses.

qPCR showed 50% reduction of full-length *Shank3 *mRNA in the heterozygotes and complete loss in knockouts (Figure 1B). Moreover, there was no expression of full-length Shank3 protein in PSD fractions from *Shank3*-knockout mice and reduced expression in the heterozygotes, using antibodies which cross-react either with an epitope downstream of the PDZ domain (antibody N69/46; see Figure 1A) (Figure 1C) or with the COOH terminal (data not shown), consistent with haploinsufficiency.

Heterozygous and homozygous animals were viable and showed no obvious alterations in gross brain structure or hippocampal cytoarchitecture, nor were there any obvious seizures. There was evidence for subtle motor abnormalities in homozygotes, which is being further characterized in ongoing studies. Both genotypes were normal on measures of general health, developmental milestones and exploratory activity, as well as on social approach as measured in an automated three-chambered social approach task.

Because *SHANK3 *haploinsufficiency is responsible for the neurobehavioral phenotype in individuals with 22q13DS or *SHANK3 *mutations, we focused the studies reported here on *Shank3 *heterozygous mice generated by crossing wild-type mice with heterozygotes to be most relevant to the clinical syndromes and to be most useful in ultimately assessing potential therapeutic interventions in preclinical studies. A comprehensive investigation of the knockout mice obtained through an alternate breeding strategy (heterozygote-heterozygote matings) is now in progress in independent experiments.

### Basal glutamatergic synaptic transmission is reduced in *Shank3 *heterozygous mice

**Figure 2:**
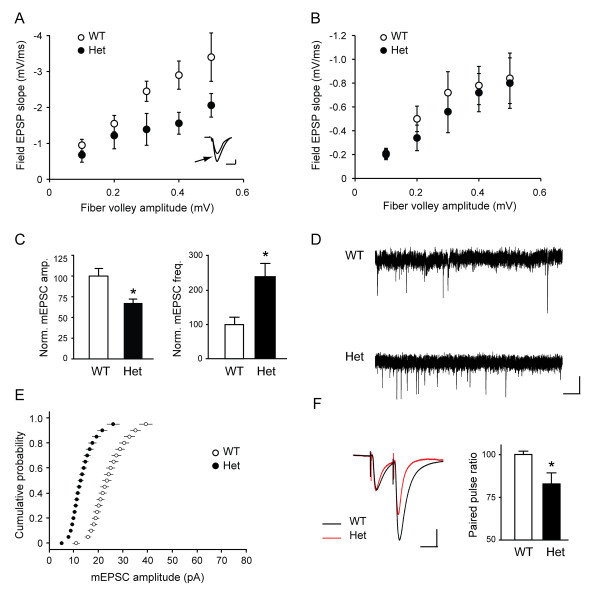
**Altered basal synaptic properties in *Shank3 *heterozygous mice**. **(A) **Reduced α-amino-3-hydroxyl-5-methyl-4-isoxazole-propionic acid (AMPA) receptor responses in *Shank3 *heterozygous mice. Slices were incubated in the presence of 2-amino-5-phosphonopentanoic acid (APV) and mean field excitatory postsynaptic potential (field EPSP) slope as a function of fiber volley is shown for slices derived from wild-type and heterozygous mice. The *inset *shows representative traces for a given stimulus intensity (0.5 mA) in the input/output (I/O) graph (arrow indicates the trace from wild-type; scale: 10 ms, 0.5 mV). **(B) **Normal *N*-methyl-D-aspartic acid (NMDA) receptor responses in *Shank3 *heterozygous mice. Slices were incubated in the presence of 6-cyano-7-nitroquinoxaline-2,3-dione (CNQX), and mean field EPSP slope as a function of fiber volley is shown. **(C) **Miniature excitatory postsynaptic currents (mEPSCs) from wild-type and *Shank3 *heterozygous mice. *Left*: Amplitude of mEPSCs. *P < 0.01. *Right*: Frequency of mEPSCs. *P < 0.03. **(D) **Sample traces of mEPSCs. Scale: 1 s, 40 pA. **(E) **Cumulative probability of mEPSC amplitude. **(F) **Paired-pulse ratio. *Left*: Representative EPSC traces from *Shank3 *heterozygous (red) and wild-type (black) mice, with traces normalized to the first EPSC for comparison. *P < 0.05. WT, wild-type mice; Het, *Shank3 *heterozygous mice.

To examine the role of Shank3 in regulating synaptic glutamate receptor function, we studied glutamatergic synaptic transmission in hippocampal slices. We examined the properties of basic transmission at Schaffer collateral-CA1 synapses in hippocampal slices from 3- to 4-week-old *Shank3 *heterozygous mice and their littermates using extracellular recordings. Plotting field excitatory postsynaptic potential (fEPSP) slope versus stimulus intensity demonstrated a reduction in the I/O curves in the heterozygotes (data not shown), prompting us to then further examine synaptic transmission in the presence of inhibitors of specific subtypes of glutamate receptors. In the presence of the NMDA receptor antagonist APV, a decrease in AMPA receptor-mediated field potentials in the heterozygous mice was seen, reflected as a 50% decrease in the average slope of I/O function compared with wild-type mice (*n *= 4 mice per genotype, two to three slices per mouse; *P *= 0.001; Figure 2A). In contrast, when the I/O relationship was analyzed in the presence of the competitive AMPA/kainate receptor antagonist CNQX to measure synaptic NMDA receptor function, there was no difference between genotypes (*n *= 4 mice per genotype, two to three slices per mouse; *P *= 0.1; Figure 2B). These results indicate that there is a specific reduction in AMPA receptor-mediated basal transmission in the *Shank3 *heterozygous mice.

We asked whether the impairment of evoked synaptic transmission in heterozygous mice is caused by alterations in presynaptic and/or postsynaptic parameters. We performed whole cell patch-clamp recordings in 3-month-old littermates and monitored spontaneous miniature postsynaptic currents in the presence of tetrodotoxin (TTX). The amplitude of miniature excitatory postsynaptic currents (mEPSCs) from hippocampal CA1 pyramidal neurons of heterozygous mice were significantly smaller than those in control mice (*n *= 7 for wild-type and *n *= 8 for heterozygous mice, *P *< 0.01; Figures 2C and 2D), which was evident by the significant shift of the cumulative probability to the left (Figure 2E), again indicating a reduction in basal transmission. However, in heterozygous mice, the frequency of miniature excitatory postsynaptic currents was significantly higher (*n *= 7 for wild-type and *n *= 8 for heterozygous mice, *P *< 0.03; Figures 2C and 2D) and paired-pulse ratio was decreased (*n *= 6 for wild-type and *n *= 7 for heterozygous mice, *P *< 0.05; Figure 2F), which revealed an additional, presynaptic alteration in the heterozygotes as well.

### Long-term potentiation is impaired in *Shank3 *heterozygous mice

**Figure 3:**
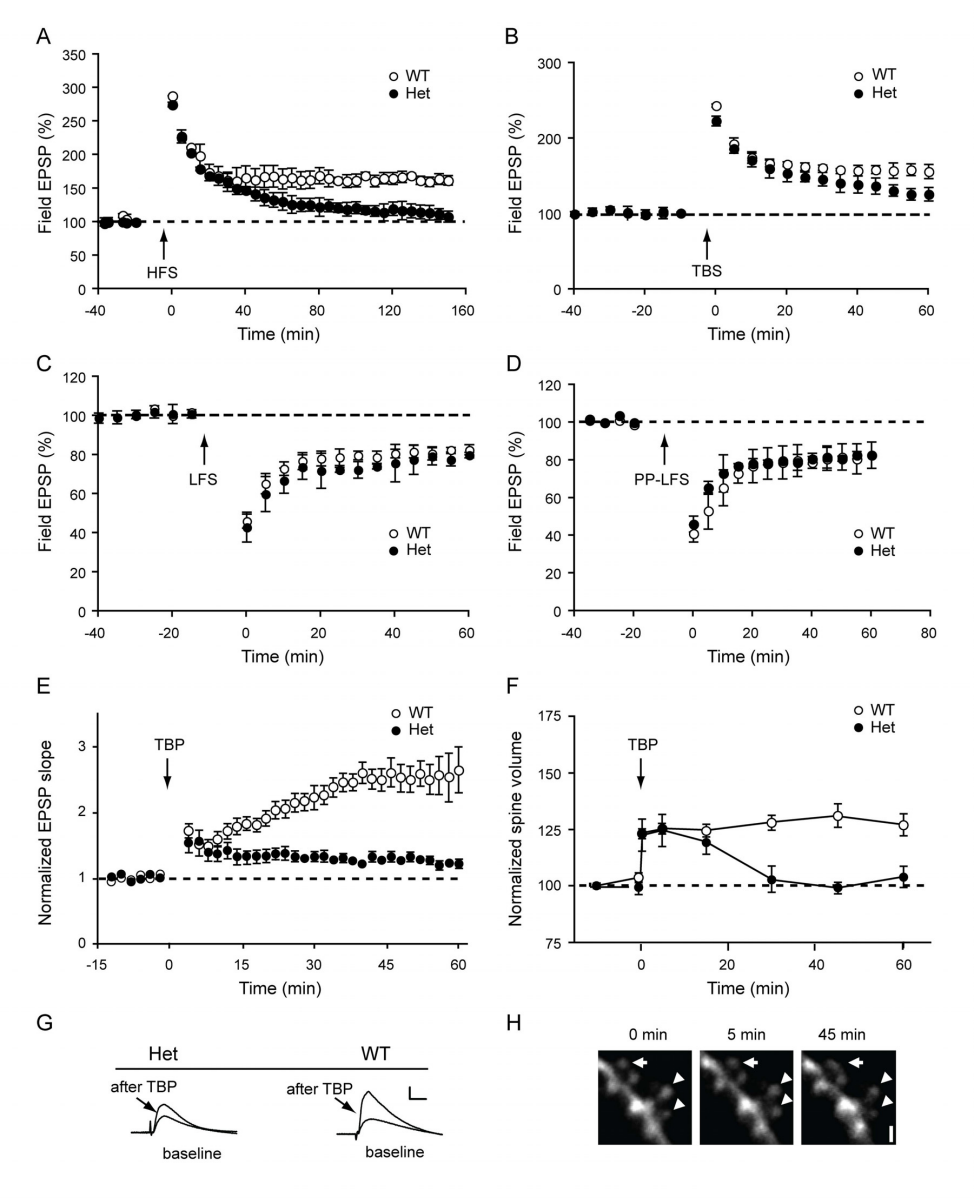
**Reduced long-term potentiation in *Shank3 *heterozygous mice**. **(A) **Long-term potentiation (LTP) following high-frequency stimulation. Field recordings of LTP induced with high-frequency stimulation (HFS; 4 times 100 Hz, separated by 5 min) as a function of time in slices from wild-type and *Shank3 *heterozygous mice. (B) LTP following θ-burst stimulation (TBS). **(C) **Long-term depression (LTD) following low-frequency stimulation (LFS), an NMDA receptor-dependent form of LTD. **(D) **LTD following paired-pulse low-frequency stimulation (PP-LFS), a protein synthesis-dependent form of LTD. **(E) **LTP recorded with whole cell patch-clamp method. LTP was induced with θ-burst pairing (TBP). **(E) **Normalized EPSP slope is shown as a function of time. **(G) **Representative EPSP traces before and after (arrow) LTP induction (scale bar: 5 mV, 10 ms). **(F) **Changes in normalized spine volume following LTP. **(H) **Representative images showing TBP-induced spine expansion in *Shank3 *heterozygotes. Images were acquired before and 5 min and 45 min after TBP. Transiently increased and stable spines are indicated by arrowheads and arrow, respectively (scale bar: 1 μm). WT, wild-type mice; Het, *Shank3 *heterozygous mice.

We next examined long-term potentiation (LTP) with extracellular fEPSP recordings at Schaffer collateral/CA1 synapses. In the first set of experiments, LTP was induced by tetanic stimulation of the Schaffer collaterals (four trains of 100 Hz separated by 5 min). While initial expression of LTP was identical across the two genotypes, the maintenance of LTP was clearly impaired in the heterozygous mice (average percentage of baseline 120 min after tetanus: 165.1 ± 8.8% in wild-type and 117.1 ± 9.5% in heterozygous mice, *P *= 0.004; Figure 3A). In an additional set of experiments, we further tested TBS LTP (10 bursts of four pulses at 100 Hz separated by 200 ms), which also showed a significant decrease in the potentiation at 60 min after TBS in heterozygous mice (156.3 ± 9.2% of baseline in wild-type and 126.0 ± 8.9% in heterozygous mice, measured 60 min after TBS, *P *= 0.007; Figure 3B).

In contrast to the altered synaptic plasticity observed with LTP, long-term depression (LTD) induced by either low-frequency stimulation (LFS) (82.6 ± 1.35% of baseline in wild-type and 79.9 ± 2.5% in heterozygous mice, measured 60 min after LFS, *P *> 0.1; Figure 3G) or paired-pulse LFS (PP-LFS) stimulation (81.6 ± 6% of baseline in wild-type and 82.7 ± 1.9% in heterozygous mice, *P *> 0.1; Figure 3H) was not significantly changed in heterozygotes.

Previous studies have shown that LTP is accompanied by spine enlargement [35,48]. Therefore, it was of interest to determine whether the deficits in LTP in the *Shank3 *heterozygous mice were associated with altered spine remodeling. We first established that spines from wild-type mice were capable of structural modification by simultaneously monitoring spine size and synaptic responses in CA1 neurons before and after TBP [34,35]. We found that TBP produced a rapid and persistent increase in spine volume concurrent with an immediate increase in EPSP slope in whole cell recordings, which gradually reached a plateau by ~30 min (Figures 3C-F). However, in recordings from heterozygous mice, we found that the stabilization in synaptic potentiation and spine expansion were impaired in CA1 neurons (Figures 3C-F). In the heterozygous mice, both EPSP slope and spine volume increased immediately to values comparable to those of control spines, but synaptic potentiation and spine expansion failed to be sustained.

### GluR1 immunoreactivity is decreased in *Shank3 *heterozygous mice

**Figure 4:**
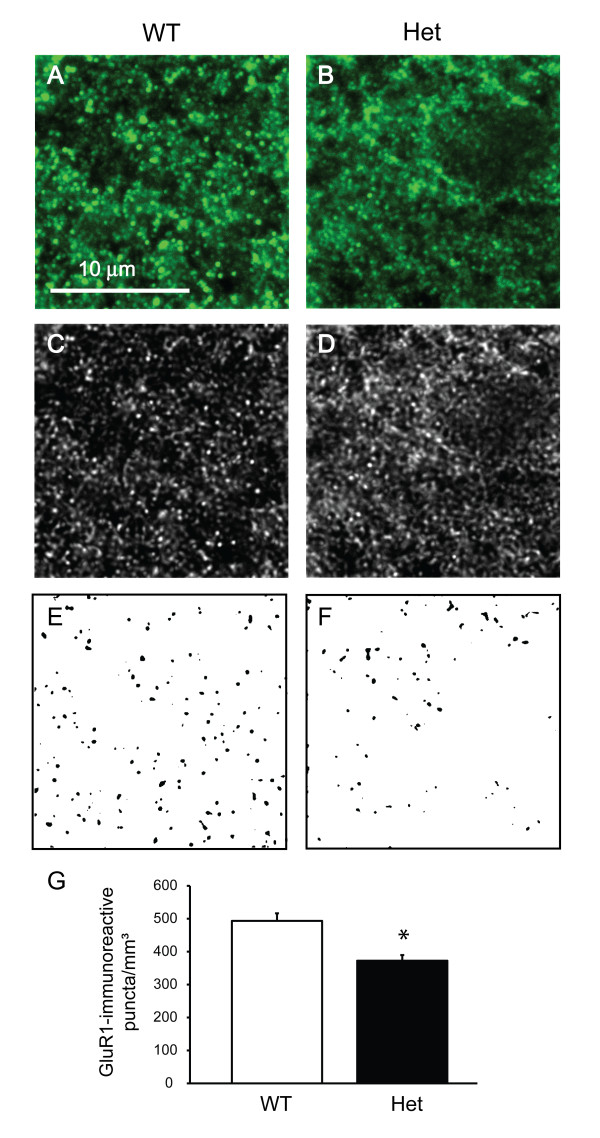
**Decreased density of GluR1-immunoreactive puncta in *Shank3 *heterozygous mice**. **(A **and **B) **High-resolution confocal images of puncta from wild-type and heterozygous mice. **(C **and **D) **The same images from Figures 4A and 4B are shown after deconvolution. **(E **and **F) **The puncta were quantified after thresholding of fluorescence intensity. **(G) **Quantification of GluR1-immunoreactive puncta. *P < 0.005. WT, wild-type mice; Het, *Shank3 *heterozygous mice.

We then asked whether *Shank3 *haploinsufficiency can lead to alterations in the numbers of AMPA receptor-positive puncta, given the results from the electrophysiological experiments and the relationship between synaptic strength and AMPA receptor subunit trafficking [49]. We carried out immunolabeling for GluR1 (an AMPA receptor subunit) and quantified GluR1-immunoreactive puncta (Figures 4A-G). Neurons from *Shank3 *heterozygous mice showed significantly fewer GluR1-immunoreactive puncta (*P *< 0.005) consistent with the electrophysiological data.

### Behavioral analyses of *Shank3 *heterozygotes

**Figure 5:**
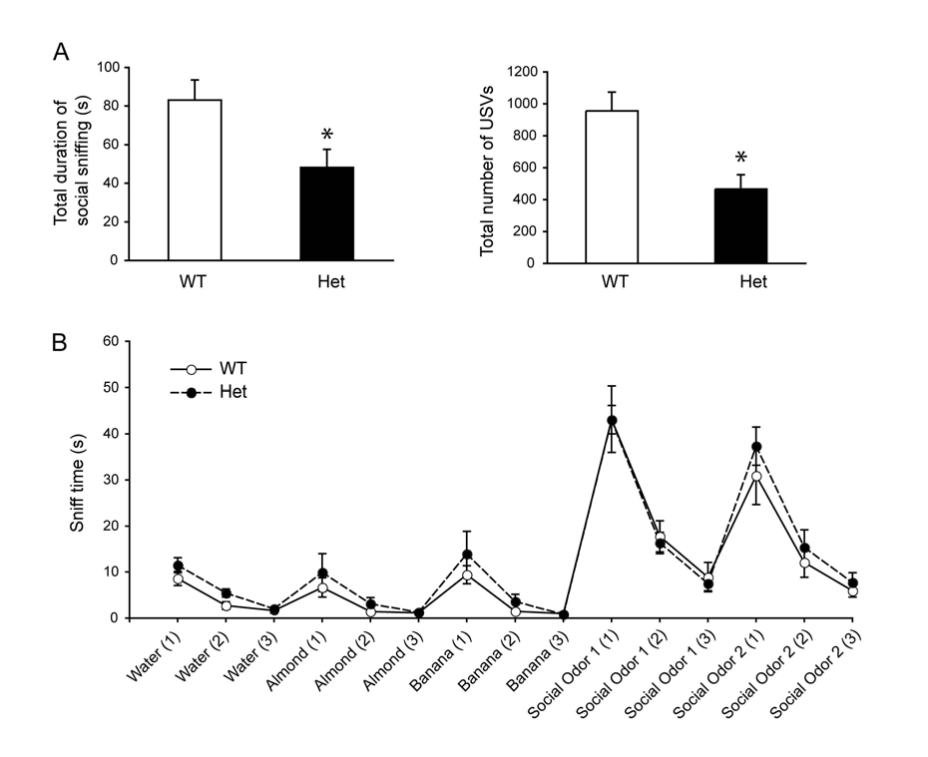
**Reduced social behaviors in *Shank3 *heterozygous and wild-type littermate mice**. **(A) **Adult male-female social interactions. *Left*: Total duration of social interactions, scored as cumulative seconds spent by the male subject in sniffing the nose, anogenital and other body regions of an unfamiliar adult estrus B6 female mouse during a 5-min test session in a clean, empty mouse cage. *P = 0.02. *Right*: Number of ultrasonic vocalizations emitted during the social interaction test session (*P = 0.003) (*n *= 12 for wild-type (WT) and *n *= 14 for heterozygous (Het) mice). **(B) **Olfactory habitutation and dishabituation to nonsocial and social odors, measured as cumulative time spent sniffing a sequence of identical and novel odors delivered on cotton swabs inserted into a clean cage (*n *= 8 mice/genotype).

To more extensively study social interactions in *Shank3 *heterozygous mice, we examined male-female social interactions during a 5-min session of freely moving reciprocal social interactions with an estrus B6 female (Figure 5A). Cumulative duration of total social sniffing by the male test subjects was lower in *Shank3 *heterozygotes than in wild-type littermates (*P *= 0.02). In addition, fewer ultrasonic vocalizations were emitted by heterozygotes than by wild-type littermates during the male-female social interaction session (*P *= 0.003). Note that while the equipment used could not distinguish between calls emitted by the male subject and female partner, the preponderance of calls during male-female interactions in mice is usually emitted by the male [50].

As a control task to ensure that subject mice can detect the social pheromones that elicit approach and vocalizations, we measured olfactory abilities using the olfactory habituation/dishabituation test. Habituation and dishabituation were normal for social and nonsocial odor cues in both wild-type and *Shank3 *heterozygous mice (*n *= 8/genotype; Figure 5B). Both genotypes displayed the expected habituation, indicated by decreased time spent in sniffing the sequence of three same odors, and the expected dishabituation, indicated by increased time sniffing the different odor (water habituation, wild-type: *P *< 0.001; water habituation, heterozygotes: *P *< 0.001; almond habituation, wild-type: *P *= 0.015; almond habituation, heterozygotes: *P *= 0.04; banana habituation, wild-type: *P *< 0.001; banana habituation, heterozygotes: *P *= 0.033; social odor 1 habituation, wild-type: *P *< 0.001; social odor 1 habituation, heterozygotes: *P *< 0.001; social odor 2 habituation, wild-type: *P *< 0.001; social odor 2 habituation, heterozygotes: *P *< 0.001; water to almond dishabituation, wild-type: *P *= 0.061; water to almond dishabituation, heterozygotes: *P *= 0.118; almond to banana dishabituation, wild-type: *P *= 0.005; almond to banana dishabituation, heterozygotes: *P *= 0.046; banana to social odor 1 dishabituation, wild-type: *P *< 0.001; banana to social odor 1 dishabituation, heterozygotes: *P *= 0.046; social odor 1 to social odor 2 dishabituation, wild-type: *P *< 0.001; social odor 1 to social odor 2 dishabituation, heterozygotes: *P *< 0.001).

## Discussion

In the present study, we summarize our results in mice with a disruption of the full-length Shank3 protein. We observed reductions in glutamatergic synaptic transmission and plasticity, including deficits in AMPA receptor-mediated transmission and spine remodeling. We also identified a reduced number of GluR1-immunoreactive puncta in the striatum radiatum. Finally, we saw evidence of social interaction and social communication deficits in these mice. These results demonstrate the importance of *Shank3 *in synaptic function, ultimately leading to behavioral changes, which may be relevant to the symptoms in individuals with *SHANK3 *mutations.

### Development of the *Shank3*-deficient model

Our study targeted the full-length reference *Shank3*. A recent report has indicated that there are additional start sites in the human and mouse *SHANK3 *gene that may lead to shorter products, truncated at the N terminus (termed 22t and 32t in that report [51]). It is therefore possible that our *Shank3*-deficient mice are still expressing shorter forms of Shank3 protein. We examined the possibility that there were high levels of such shorter forms of Shank3 in mouse brain, making use of an antibody with an epitope downstream of the PDZ domain (antibody N69/46; see Figure 1A) that should recognize both the longer form and the predicted shorter (22t and 32t) forms (if expressed in the same frame as the longer form) (see transcripts labeled 22t and 32t in Figure 1A). In PSD fractions (see Figure 1) and in mouse brain extracts [52], we saw strong expression of only a single form and little evidence for high expression of shorter forms. However, the caveat remains that all deficits seen with deletion of the entire *SHANK3 *gene (including 22t and 32t forms) may not be fully recapitulated in the model presented here. We do note that a *de novo *mutation in the ankyrin repeat domain has been reported in a child with an ASD [27], providing strong support for a role for loss of the longer ankyrin repeat domain-containing form in neurodevelopmental disabilities. Hence, disruption of this form, as done in the current study, has direct construct validity to ASD and to neurodevelopmental disabilities. It will be of interest to determine where and to what levels the 22t and 32t forms are expressed and to assess whether neuronal and/or other phenotypes are associated with their disruption. Careful characterization of mice with a disruption of *Shank3 *by targeting downstream exons common to the full-length, 22t and 32t forms will be of great interest, with the caveat that such targeting may produce dominant-negative shorter products truncated at the C-terminal that, depending on the exons targeted, may or may not be relevant to known clinical mutations.

### Synaptic development and function in *Shank3 *heterozygotes

Electrophysiological studies of the mice showed a decrease in basal synaptic transmission in heterozygotes when assessed by comparing either the I/O relationship using field recordings or the amplitude of miniature excitatory postsynaptic currents (mEPSCs) using patch clamp recordings. This reduction in AMPA receptor-dependent transmission is likely mediated by a decrease in the number of synaptic AMPA receptors. *Shank3 *heterozygous mice also showed a decrease in the density of GluR1-immunoreactive puncta, consistent with a reduction in AMPA receptor levels. On the other hand, both increase in mEPSC frequency and decrease in paired pulse ratio in *Shank3 *heterozygous mice were consistent with an increase in presynaptic release.

Transfection of *Shank3 *into aspiny cerebellar granule cells has been shown to induce an increase in the AMPA component of mEPSCs [19], and Shank3 is colocalized with GluR1-containing AMPA receptor in typical spines [53]. In our studies, haploinsufficiency of Shank3 *in vivo *leads to reduced levels of AMPA receptors and GluR1-positive puncta, results which are consistent with such studies *in vitro*. However, while the postsynaptic alterations in the *Shank3*-deficient mice are consistent with studies carried out *in vitro*, understanding the changes in presynaptic function observed in our analyses will need further investigation. This presynaptic alteration could be explained by compensatory mechanisms in the *Shank3 *heterozygous mice to maintain constant synaptic function. However, this compensatory effect falls short as evidenced by the reduction in the I/O relationship.

Selective acquisition of AMPA receptors is thought to be a crucial mechanism for synapse maturation and function, and our data confirm that Shank3 is an important mediator of AMPA receptor recruitment and of synaptic development.

### Synaptic plasticity and spine remodelling in *Shank3 *heterozygotes

We examined LTP induced by either high frequency stimulation or TBS. In *Shank3 *heterozygous mice, reduced LTP was apparent at 30 min after induction in either paradigm and no potentiation was observed at 120 min after induction. However, the initial expression of LTP was normal with either induction paradigm. We observed similar deficits in the spine expansion that normally accompanies LTP, such that the initial expansion was normal in heterozygotes but quickly decayed to baseline. The simplest explanation for these deficits is an alteration in the trafficking of AMPA receptors to synapses. AMPA receptor trafficking is involved in activity-induced changes in synaptic strength associated with learning and memory [54], and synaptic insertion of GluR1-containing AMPA receptors is required [55,56]. The quantity of surface receptors controls synaptic strength [57-59]; therefore, reduction of both synaptic transmission and plasticity in the *Shank3 *heterozygotes would support a mechanism involving altered AMPA receptor trafficking. Initial LTP is likely mediated by phosphorylation of AMPA receptors, and the initial spine expansion occurs in the absence of trafficking of AMPA receptors [54]. However, AMPA receptor trafficking to the synapse is critical to sustain LTP and spine expansion. The transient nature of synaptic potentiation and spine enlargement in heterozygous mice is consistent with normal phosphorylation of existing synaptic AMPA receptors with a deficit in subsequent consolidation, the process in which both structural and functional forms of plasticity are progressively stabilized over 30 min [34].

### Social behavior in *Shank3 *heterozygotes

Male *Shank3 *heterozygous mice displayed reduced responses to female social cues compared to their wild-type littermates on parameters of social sniffing and ultrasonic vocalizations in a male-female reciprocal social interaction context. Given that Shank3 has a central role in synaptic function, Shank3-related changes in cellular and network components that underlie social cognition could underlie the reduced social interactions in *Shank3 *heterozygous mice. It was interesting that social deficits were not observed in a three-chamber social approach task, implying some specificity of the social alterations. It will be of interest to better understand the frequency of ASD and the characteristics of social deficits in *SHANK3*-haploinsufficiency syndromes, as it is already evident that this is not a universal finding in these syndromes when considering 22q13DS or schizophrenia.

The social deficits observed in the mice serve as important reminders that systems outside the hippocampus that are involved in social behaviors will need to be examined in these mice and that additional behaviors known to be mediated by the hippocampus will be important for further investigations. Cellular and electrophysiological analyses in additional brain regions and large-scale behavioral studies are now underway for the *Shank3*-deficient mice in the authors' laboratories.

## Conclusions

Haploinsufficiency of full-length *Shank3 *resulted in a decrease in synaptic transmission, altered functional and structural plasticity of synapses and reduced social behaviors. These findings are consistent with a model of delayed synaptic development in *Shank3 *haploinsufficiency, together with a reduction in AMPA receptor trafficking. The results highlight the importance of Shank3 in synaptic development and function and support a link between deficits in synapse function and neurodevelopmental disorders. To date, pharmacological treatments for ASDs and other developmental disorders (including *SHANK3*-haploinsufficiency syndromes) are primarily ameliorative, focusing on managing associated systems such as anxiety, aggression, repetitive behaviors, attention deficits and epilepsy, among others (see, for example, [60]). Pharmacological treatments addressing core diagnostic symptoms, including, in ASDs, alterations in reciprocal social interactions and communication, do not yet exist. Recently, the field has begun to see the evaluation of therapies targeted to etiology (that is, "personalized medicine") using models of neurodevelopmental disorders including fragile X, tuberous sclerosis, and Rett syndromes (see, for example, [61-64]). The use of model systems such as the *Shank3*-deficient mice reported here could lead to similar advances in the case of *SHANK3*-haploinsufficiency syndromes. One interesting outcome of the current study is that compounds that enhance glutamatergic transmission, including those that specifically enhance AMPA transmission (AMPAkines) could possibly represent therapeutic approaches in these conditions. Further analysis of this and additional models may identify targets for novel therapeutics for individuals with developmental delays arising from 22q13 deletion syndrome or *SHANK3 *mutations.

## Competing interests

OB, TS and JDB have submitted a patent on this work.

## Authors' contributions

TS and JDB generated and biochemically characterized the mice. JDB, PRH, JNC, TS, QZ and OB designed the experiments. OB, XW and QZ performed the electrophysiology experiments and analysis. Immunohistochemistry as well as confocal microscopy and analysis were conducted by DP, DLD and PRH. Behavioral experiments and analysis were conducted by MY, AMK, MLS, MJH, RS, JLS and JNC. qPCR was conducted by NT. Immunoblotting was conducted by YK. The manuscript was written by OB and JDB, and all authors reviewed the manuscript before submission.
